# Scrotal metastases from colorectal carcinoma: a case report

**DOI:** 10.1186/1757-1626-2-111

**Published:** 2009-01-31

**Authors:** Doireann M McWeeney, Sean T Martin, Ronan S Ryan, Iqdam N Tobbia, Paul P Donnellan, Kevin M Barry

**Affiliations:** 1 Department of Surgery, Mayo General Hospital, Castlebar, Co. Mayo, Ireland; 2 Department of Radiology, Mayo General Hospital, Castlebar, Co. Mayo, Ireland; 3 Department of Pathology, Mayo General Hospital, Castlebar, Co. Mayo, Ireland; 4 Department of Medical Oncology, Mayo General Hospital, Castlebar, Co. Mayo, Ireland

## Abstract

A 72-year-old man presented with a two month history of rectal bleeding. Colonoscopy demonstrated synchronous lesions at 3 cm and 40 cm with histological analysis confirming synchronous adenocarcinomata. He developed bilobar hepatic metastases while undergoing neoadjuvant chemoradiotherapy. Treatment was complicated by Fournier's gangrene of the right hemiscrotum which required surgical debridement. Eight months later he re-presented with an ulcerating lesion on the right hemiscrotum. An en-bloc resection of the ulcerating scrotal lesion and underlying testis was performed. Immunohistological analysis revealed metastatic adenocarcinoma of large bowel origin. Colorectal metastasis to the urogenital tract is rare and here we report a case of rectal carcinoma metastasizing to scrotal skin.

## Background

Colorectal cancer is the second commonest non-cutaneous cancer diagnosed in the Republic of Ireland, with over 1800 new cases annually. At presentation, approximately 35% of patients have advanced disease [1]. Occasionally, patients will present with metastatic deposits in unusual sites including the skin [2]. Metastases to the urogenital tract have been reported in the literature, more frequently in males [3], but also in females [4]. Here we describe a 72 year-old male with low rectal cancer who developed metastases to the scrotal skin.

## Case presentation

A 72-year-old Caucasian man presented in January 2006 with a two-week history of symptomatic hypochromic, microcytic anaemia of 8 g/dL. He gave a short history of rectal bleeding and tenesmus. Colonoscopy demonstrated an exophytic, circumferential, near-obstructing lesion 3 cm from the anal verge with a synchronous exophytic lesion in the sigmoid colon. Biopsies confirmed the presence of synchronous poorly-differentiated adenocarcinomata of these sites.

Radiological staging with endo-anal ultrasound demonstrated a 2.2 cm diameter mass lesion in the rectum extending beyond the rectal wall. Magnetic resonance imaging (MRI) of anorectum confirmed a locally advanced neoplasm with suspicion of involvement of one mesorectal lymph node- Staging pT3N1Mx. Computed tomography (CT) of abdomen and pelvis confirmed a locally-invasive distal rectal neoplasm with no evidence of distant metastases.

Due to the development of obstructive symptoms, the patient required a laparoscopic defunctioning loop ileostomy prior to commencing neoadjuvant chemoradiotherapy. Two weeks post-operatively he developed Fournier's gangrene of the right hemiscrotum which was successfully treated by surgical debridement, delaying the commencement of neoadjuvant therapy.

Pre-operative chemoradiotherapy consisted of 5,040 centiGray (cGy) delivered in fractions of 180 cGy per day, five days per week, and 5 Fluoro-uracil given in a 120 hour continuous intravenous infusion at a dose of 1,000 mg per square metre of body surface area per day during the first and fifth weeks of radiotherapy. He was scheduled for low anteriorresection six weeks following the completion of chemoradiotherapy.

During week five of treatment, July 2006, he was noted to have deranged liver enzymes. Ultrasound scan of abdomen noted suspicious bilobar liver lesions and tri-phasic CT of liver was consistent with the development of significant bilobar liver metastases.

He was subsequently treated with the FOLFIRI regimen of palliative chemotherapy (Folic acid, 5-Fluoro-uracil and Irinotecan). Baseline CT of abdomen and pelvis in November 2006 showed progression of hepatic metastases.

**Figure 1:**
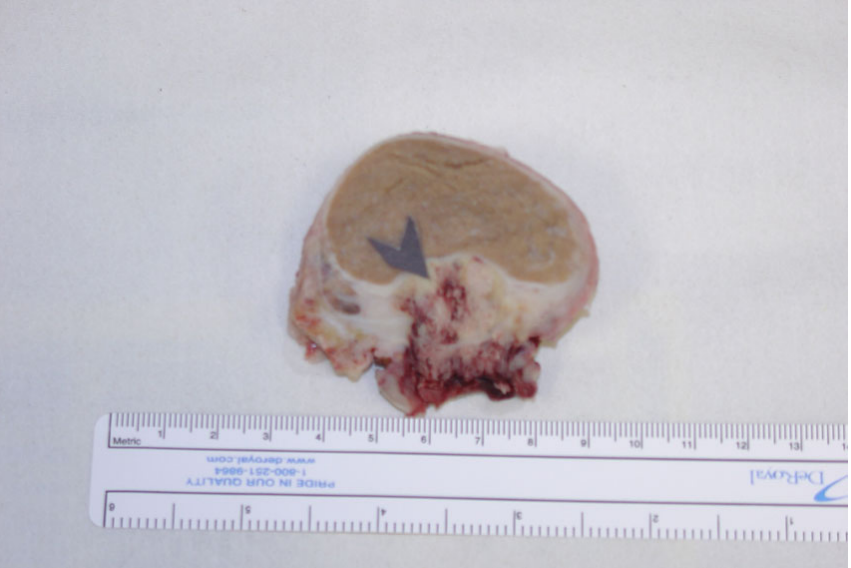
**Macroscopic appearance of excised right testis and overlying scrotal skin**. Arrow demonstrates cutaneous invasion by necrotic carcinoma.

In December 2006 he presented acutely with a painless necrotic ulcer affecting the skin of the anterior right hemiscrotum. Ultrasound of scrotum demonstrated a heterogenous solid vascular lesion of the scrotal skin thought to originate in the upper pole of the right testis (figure 1).

**Figure 2:**
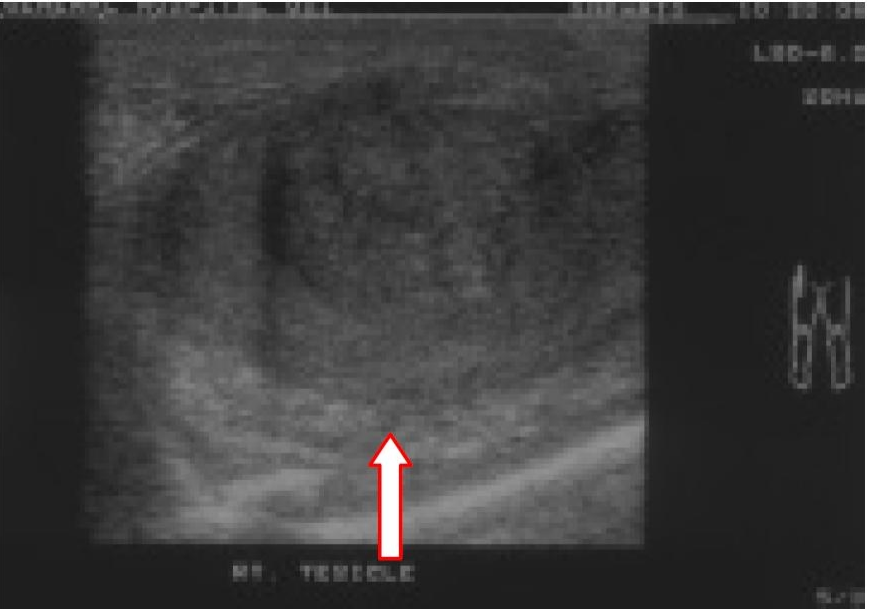
**Ultrasound right testis demonstrating loss of tissue plane between testis and adjacent structures/overlying skin (arrow)**.

**Figure 3:**
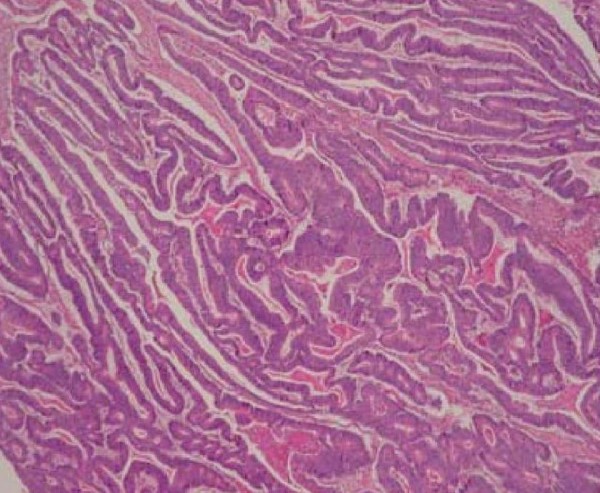
**Haematoxylin and eosin (H&E) stains positive for carcinoma cells, magnification × 40**.

He underwent radical en-bloc resection of the scrotal skin and underlying testis due to intra-operative suspicion of testicular involvement (figure 2). Histological analysis of the excised specimen showed metastatic moderately differentiated adenocarcinoma, most likely of large bowel origin, infiltrating the skin and subcutaneous tissue of the scrotum (figure 3).

**Figure 4:**
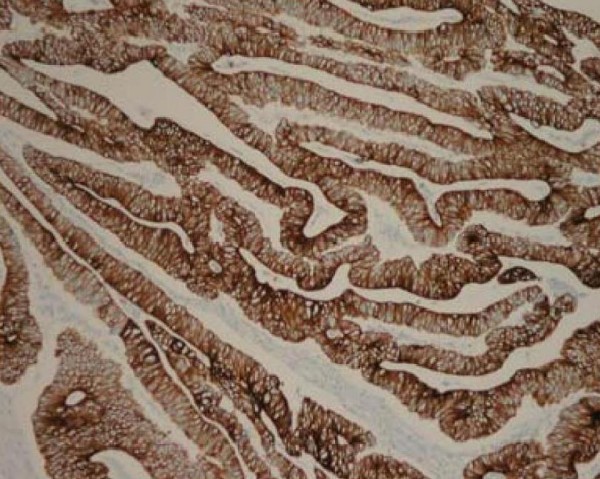
**Demonstrates tumour tissue staining strongly positive for CEA, magnification × 400**.

**Figure 5:**
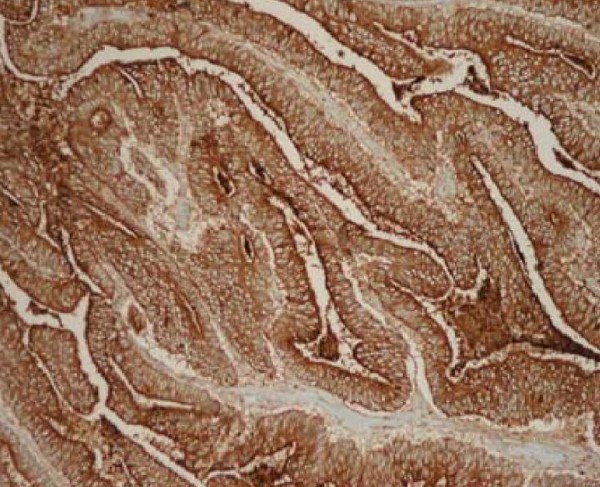
**Demonstrates tumour tissue staining strongly positive for CK-20, magnification × 400**.

Immunohistochemical staining of tumour tissue was positive for carcinoembryonic antigen (CEA) (figure 4) and cytokeratin 20 (CK-20) (figure 5) indicating that the malignant cells were of colonic origin.

## Conclusion

Cutaneous colorectal metastases are rare and signify advanced disease. Metastases in incision sites typically occur in the incision made at time of cancer resection surgery rather than in a pre-existing scar [5]. Abdominal wound metastases are the most frequent site of cutaneous metastasis from colorectal adenocarcinoma, although cutaneous metastases at other sites have been reported including the extremities, genitalia, head and neck [6,7]. Several mechanisms of cutaneous metastases have been postulated including haematogenous spread, lymphatic spread, direct extension, spread along ligaments of embryonic origin and seeding of exfoliated tumour cells. Tumour cell seeding or implantation is thought to be the most likely pathogenesis where there is metastasis to incisions made at laparoscopic or open colectomy for colorectal cancer resection. We postulate that the propensity for incisional metastasis results from changes in the local cellular environment, for example altered lymphatic drainage or increased propensity for tumour cell deposition secondary to modified adhesion molecule profile or defective host surveillance. The definitive pathogenesis of incisional metastasis remains a matter of conjecture and warrants further evaluation in the in-vivo and in-vitro setting.

## Consent

Written informed consent was obtained from the patient's next or kin for publication of this case report and accompanying images. A copy of the written consent is available for review by the Editor-in-Chief of this journal.

## Competing interests

The authors declare that they have no competing interests.

## Authors' contributions

DM reviewed the case history and prepared the case report. SM reviewed the literature and prepared the final draft. KB, SM, and DM were involved in the surgical care of the patient. RR interpreted the radiological images. IT performed the histological examination of the scrotal specimen and immunohistochemical staining and analysis. PD was involved in oncological care. All authors read and approved the final manuscript.
